# Primary Ovarian Neuroendocrine Neoplasm With Carcinoid Syndrome and Carcinoid Heart Disease

**DOI:** 10.31486/toj.25.0101

**Published:** 2026

**Authors:** Kitti Thammakosol, Prin Vathesatogkit, Surakiat Leelasithorn, Chutintorn Sriphrapradang

**Affiliations:** ^1^ Division of Endocrinology and Metabolism, Department of Medicine, Faculty of Medicine Ramathibodi Hospital, Mahidol University, Bangkok, Thailand; ^2^ Chakri Naruebodindra Medical Institute, Faculty of Medicine Ramathibodi Hospital, Mahidol University, Samut Prakan, Thailand; ^3^ Division of Cardiology, Department of Medicine, Faculty of Medicine Ramathibodi Hospital, Mahidol University, Bangkok, Thailand

**Keywords:** *Carcinoid heart disease*, *carcinoid tumor*, *malignant carcinoid syndrome*, *octreotide*, *ovarian neoplasms*

## Abstract

**Background:**

Neuroendocrine neoplasms commonly arise in the midgut, lungs, or pancreas, while primary ovarian neuroendocrine neoplasms are rare and can present with carcinoid syndrome and carcinoid heart disease even in the absence of hepatic metastases.

**Case Report:**

A 70-year-old female with coronary artery disease presented with 3 months of dyspnea, leg edema, diarrhea, and facial flushing. Examination suggested right-sided heart failure with a palpable lower abdominal mass. Her 24-hour urinary 5-hydroxyindoleacetic acid level was markedly elevated (106.0 mg), and serum chromogranin A was also elevated (468.6 ng/mL). Echocardiography showed severe tricuspid and pulmonic regurgitation from thickened, immobile leaflets. Imaging identified a DOTATATE-avid left adnexal mass on Gallium-68 DOTATATE positron emission tomography/computed tomography, consistent with primary ovarian neuroendocrine neoplasm. Preoperative management included long-acting octreotide, niacin, and guideline-directed heart failure therapy, followed by tumor resection. Histopathology confirmed insular-type ovarian neuroendocrine neoplasm without invasion or metastasis. Postoperatively, the patient improved clinically, and her biochemical markers normalized.

**Conclusion:**

Primary ovarian neuroendocrine neoplasms can cause carcinoid syndrome and right-sided valvulopathy without liver metastasis, likely because ovarian venous drainage bypasses hepatic first-pass metabolism. Multidisciplinary care enables prompt diagnosis, preoperative stabilization, definitive resection, and coordinated surveillance.

## INTRODUCTION

Neuroendocrine neoplasms predominantly originate in the midgut (the small intestine, appendix, and proximal colon), as well as in the lungs and pancreas.^1^ In contrast, primary ovarian neuroendocrine neoplasms are rare, accounting for <0.1% of all ovarian neoplasms.^2^ These tumors may occur as pure neuroendocrine entities or in association with ovarian teratomas.^3^

Carcinoid syndrome, a paraneoplastic syndrome resulting from the systemic release of vasoactive substances such as serotonin, catecholamines, tachykinins, bradykinin, kallikreins, histamine, and prostaglandins, affects approximately 20% of patients with neuroendocrine neoplasms.^4,5^ Typically, these bioactive compounds are metabolized in the liver and lungs; however, in the presence of extensive hepatic metastases or impaired metabolic clearance, they may enter the systemic circulation, leading to characteristic symptoms including cutaneous flushing, secretory diarrhea, bronchospasm, mesenteric fibrosis, niacin deficiency, and right-sided heart failure.^5,6^

Primary ovarian neuroendocrine neoplasms and neuroendocrine neoplasms with bulky retroperitoneal lymph node involvement may present with carcinoid syndrome even in the absence of hepatic metastases. This phenomenon is attributed to the direct drainage of vasoactive substances into the systemic circulation via pelvic and retroperitoneal lymphatic channels, allowing these vasoactive substances to bypass the hepatic portal circulation.^6,7^ Consequently, patients with ovarian neuroendocrine neoplasms may develop symptoms of carcinoid syndrome earlier in the disease course than patients with neuroendocrine neoplasms located in more common sites such as the midgut because hormonally active substances can enter the systemic circulation even in the absence of liver involvement.^7^

We report a rare case of a primary ovarian neuroendocrine neoplasm presenting with right-sided heart failure attributable to carcinoid heart disease.

## CASE REPORT

A 70-year-old female presented to the outpatient department with progressive exertional dyspnea and intermittent bilateral lower extremity edema for 3 months. She also reported watery diarrhea (3 to 4 episodes per day), paroxysmal facial flushing, and erythema on the neck. She denied wheezing, palpitations, headaches, or fever.

Her medical history included well-controlled hypertension and hyperlipidemia, as well as coronary artery disease, for which she had undergone percutaneous coronary intervention of the left anterior descending and left circumflex arteries 6 years prior. Current medications included aspirin 81 mg daily, pitavastatin 2 mg daily, ezetimibe 10 mg daily, and azilsartan 40 mg daily. She denied smoking or alcohol consumption and had no family history of malignancy.

Six years prior, a computed tomography (CT) colonography performed for colorectal cancer screening had incidentally revealed a 5.9-cm pelvic mass, suspected to be a subserous uterine myoma, that was asymptomatic. Since then, the patient had been under routine surveillance by her gynecologist with no notable changes reported.

**Figure 1.**
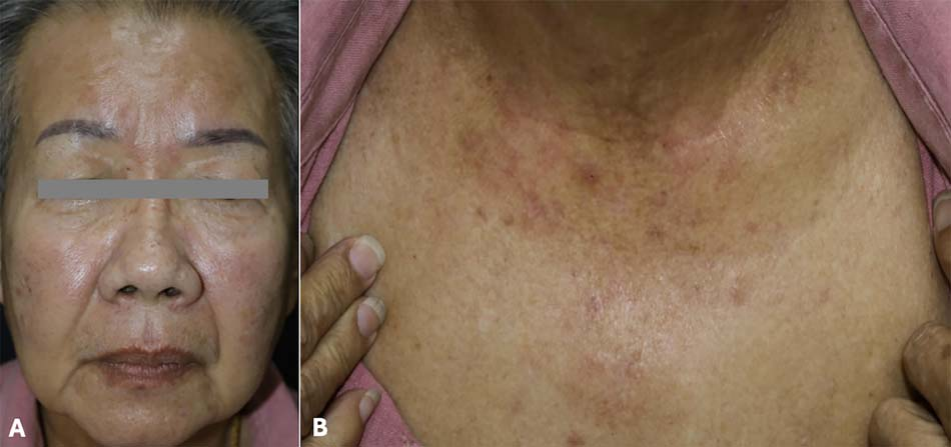
The patient presented with (A) facial flushing and (B) multiple erythematous papules and patches distributed on the anterior neck region, consistent with photosensitive dermatitis secondary to pellagra from niacin deficiency.

On physical examination, her blood pressure was 119/79 mm Hg, pulse rate was 93 beats per minute (regular), respiratory rate was 18 breaths per minute, height was 153 cm, and weight was 59 kg (body mass index 25.2 kg/m^2^). The patient had a plethoric facial appearance, with erythematous papules and patches over the anterior neck (Figure 1), and elevated jugular venous pressure with a prominent v wave. Auscultation revealed a grade 2/6 pansystolic murmur over the tricuspid area and a grade 2/6 diastolic blowing murmur over the pulmonic area. Bilateral pitting edema was noted. A firm, nontender pelvic mass approximately 10 cm in diameter was palpable in the left lower quadrant. No pulmonary crackles or wheezes were heard, and no hepatosplenomegaly or lymphadenopathy was present.

**Figure 2.**
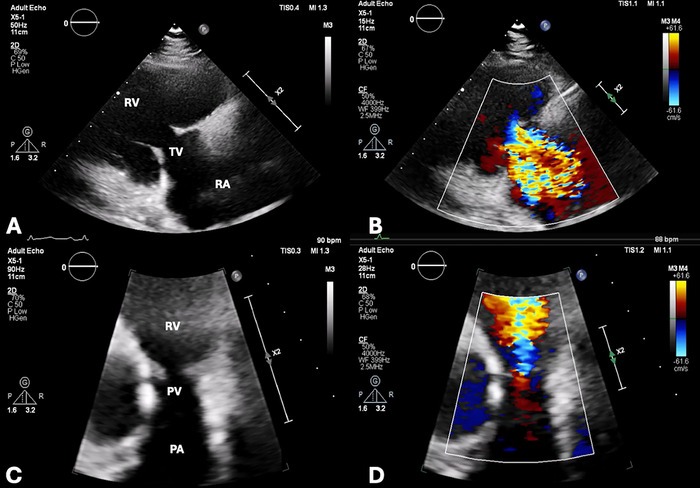
**Transthoracic echocardiogram shows (A) thickened and restricted motion of tricuspid valve (TV) leaflets in the parasternal long-axis right ventricle (RV) inflow view, (B) severe tricuspid regurgitation demonstrated by color Doppler in the parasternal long-axis RV inflow view, (C) thickened and restricted motion of the pulmonic valve (PV) cusps in the parasternal short-axis view, and (D) severe pulmonic regurgitation demonstrated by color Doppler in the parasternal short-axis view.** PA, pulmonary artery; RA, right atrium.

Transthoracic echocardiogram (Figure 2) demonstrated thickened and restricted motion of the tricuspid and pulmonic valve leaflets, causing severe noncoaptation and tricuspid regurgitation, as well as severe pulmonic regurgitation with relative pulmonic stenosis. Mild thickening of the mitral valve leaflets with trace regurgitation was also observed. The aortic valve was normal. Biventricular systolic function was preserved (left ventricular ejection fraction 58%; tricuspid annular plane systolic excursion of 1.8 cm). No pericardial effusion was noted.

Given the constellation of symptoms suggestive of carcinoid syndrome with possible carcinoid heart disease, biochemical testing was performed. The patient's 24-hour urinary 5-hydroxyindoleacetic acid level was markedly elevated at 106.0 mg (reference range, 2-9 mg), and 24-hour urinary creatinine level was 0.739 g (reference range, 0.67-1.59 g). Serum chromogranin A was elevated at 468.6 ng/mL (reference range, 0-76.3 ng/mL). The complete blood count and biochemical parameters, including liver function tests and serum electrolytes, were within normal limits.

**Figure 3.**
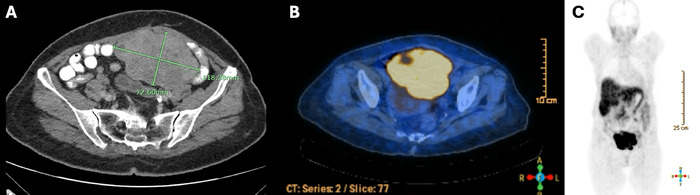
(A) Computed tomography of the abdomen shows a large, lobulated, heterogeneously enhancing mass in the left adnexa measuring 7.3 × 11.8 × 9.0 cm. (B, C) Gallium-68 DOTATATE positron emission tomography/computed tomography demonstrates intense radiotracer uptake (maximum standardized uptake value 48.9) within the adnexal lesion.

CT of the abdomen revealed a 7.3 × 11.8 × 9.0-cm lobulated, heterogeneously enhancing mass in the left adnexa, as well as a 1.3-cm subserous myoma in the right anterior uterus (Figure 3A). Gallium-68 DOTATATE positron emission tomography/CT demonstrated intense radiotracer uptake (maximum standardized uptake value 48.9) in the left adnexal mass, consistent with a somatostatin receptor–positive neuroendocrine neoplasm (Figures 3B and 3C).

The most likely diagnosis was a primary ovarian neuroendocrine neoplasm complicated by carcinoid syndrome and carcinoid heart disease, with additional findings suggestive of niacin deficiency that may have caused the erythematous patches and papules on the patient's neck.

**Figure 4.**
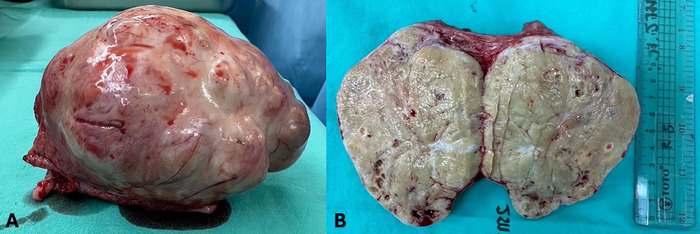
Gross appearance of the (A) resected left ovarian neuroendocrine tumor and (B) midline section showing cut surface.

The case was considered in a multidisciplinary consultation. While surgical resection was indicated, the cardiologist and endocrinologist recommended delaying the surgery to optimize cardiac status. In the interim, the patient was initiated on octreotide long-acting release (LAR) 30 mg intramuscularly every 4 weeks and oral niacin supplementation. Following clinical stabilization and administration of 2 doses of octreotide LAR, the patient underwent an elective total hysterectomy with bilateral salpingo-oophorectomy. For carcinoid crisis prophylaxis, a continuous intravenous infusion of octreotide 100 μg/h was initiated 2 hours prior to surgery and maintained for 24 hours postoperatively. The surgery was uneventful, and complete macroscopic tumor resection was achieved (Figure 4). The patient was discharged in stable condition on postoperative day 3.

Histopathologic examination confirmed an insular-type ovarian neuroendocrine neoplasm with a mitotic rate of 0-1 per 10 high-power fields. No evidence of ovarian surface involvement, lymphovascular invasion, or lymph node metastasis was seen. Immunohistochemical studies were positive for AE1/AE3, synaptophysin, and chromogranin A and were negative for estrogen receptor, progesterone receptor, calretinin, and inhibin alpha. The Ki-67 proliferation index was <1% of tumor nuclei.

At her follow-up visit, the patient reported marked improvement in symptoms including resolution of facial flushing, diarrhea, and lower extremity edema. One week after surgery and 3 weeks after the last octreotide LAR injection, the patient's 24-hour urinary 5-hydroxyindoleacetic acid and serum chromogranin A levels had decreased to 3.3 mg and 18.3 ng/mL, respectively.

## DISCUSSION

**Figure 5.**
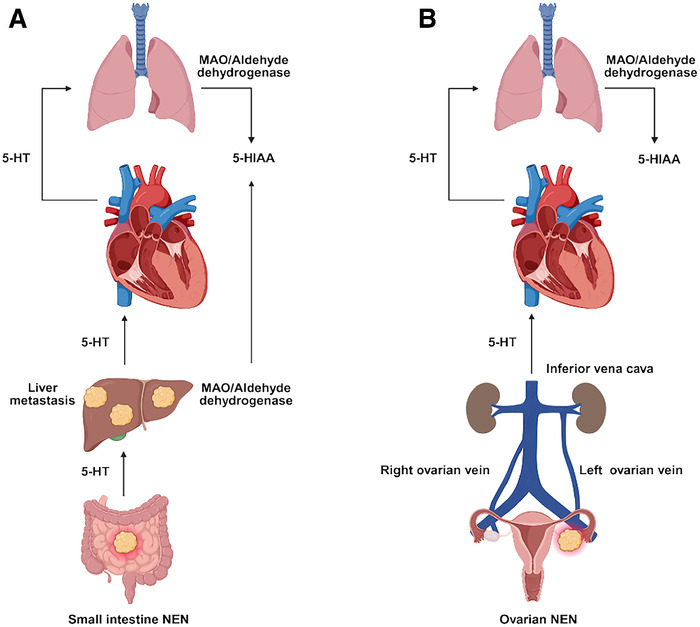
**The systemic circulation pathways of serotonin (5-hydroxytryptamine [5-HT]) that contributes to carcinoid syndrome and carcinoid heart disease are illustrated for (A) a midgut neuroendocrine neoplasm (NEN) and (B) an ovarian NEN.** 5-HIAA, 5-hydroxyindoleacetic acid; MAO, monoamine oxidase. (Figure created with BioRender.com.)

Carcinoid syndrome arising from a primary ovarian neuroendocrine neoplasm is rare. The common symptoms of facial flushing and diarrhea may mimic more common conditions such as postmenopausal syndrome and irritable bowel syndrome, often leading to delayed diagnosis.^6,8^ Unlike the typical manifestation associated with extensive hepatic metastases from midgut neuroendocrine neoplasms, ovarian neuroendocrine neoplasms bypass the hepatic portal system because of venous drainage directly into the inferior vena cava (Figure 5). This distinct drainage pathway allows vasoactive substances, particularly serotonin (5-hydroxytryptamine), to enter the systemic circulation without prior hepatic metabolism, causing carcinoid manifestations even in the absence of metastasis.^6,7,9,10^

Carcinoid heart disease is an uncommon but serious complication of carcinoid syndrome, arising from prolonged exposure to circulating serotonin.^11,12^ This exposure results in fibrous plaque formation that predominantly involves the tricuspid and pulmonary valves.^11,12^ Under normal physiology, serotonin is effectively metabolized by the liver and pulmonary vasculature; however, in the presence of hepatic metastases or when tumors bypass hepatic filtration—as observed with extrahepatic neuroendocrine neoplasms such as ovarian tumors—the right heart is subjected to unfiltered vasoactive substances. The pulmonary circulation provides partial protection to the left heart unless a right-to-left shunt, primary pulmonary tumor, or overwhelming hormone load is present. Histologically, the fibrotic plaques consist of myofibroblasts, smooth muscle cells, and extracellular matrix components, including collagen.^12^ These plaques induce thickening, stiffening, and retraction of the valve cusps, reducing their area and length, and producing combined valvular regurgitation and stenosis.^11,12^ The tricuspid valve is most frequently affected, culminating in severe regurgitation and right-sided heart failure; involvement of the pulmonary valve is common but often underestimated.^12^ Transthoracic echocardiography remains the gold standard for diagnosis, demonstrating hallmark features including thickened, immobile valve leaflets, right atrial and ventricular enlargement, subvalvular fibrosis, and characteristic Doppler flow patterns such as a triangular-shaped regurgitant systolic flow velocity profile.^13^ Mild pericardial effusion may also be observed. Advanced imaging modalities such as 3-dimensional echocardiography and cardiac magnetic resonance imaging provide enhanced anatomic and functional evaluation.^14^

Once carcinoid syndrome with multisystem involvement is established, optimal management requires a multidisciplinary approach involving cardiology, endocrinology, surgery, oncology, and anesthesiology. In our patient, although surgical excision offered curative potential, the presence of uncontrolled hormone-mediated symptoms and right-sided heart failure necessitated preoperative stabilization.^12,15^ Somatostatin analogs, such as octreotide LAR and lanreotide LAR, are cornerstone therapies for both symptom control and biochemical stabilization in patients with functioning neuroendocrine neoplasms.^12,16^ These therapies are also beneficial in delaying disease progression in metastatic settings,^16^ and their role extends to the perioperative setting to mitigate the risk of carcinoid crisis, a life-threatening complication characterized by profound hypotension, bronchospasm, and tachyarrhythmias triggered by tumor manipulation or stressors such as anesthesia and surgical intervention. Prophylaxis typically involves initiating an intravenous infusion of octreotide (50-100 μg/h) at least 2 hours prior to surgery and maintaining it for 48 hours postoperatively, followed by a gradual taper.^17-19^ Our patient received octreotide LAR 30 mg intramuscularly every 4 weeks for 2 doses, resulting in substantial clinical improvement prior to surgery. An intraoperative octreotide infusion was also administered, and the patient had an uneventful postoperative course with no evidence of carcinoid crisis or surgical complications.

The cutaneous findings observed in our patient—erythematous papules and patches—were suspected to be related to niacin deficiency (pellagra), a recognized complication of carcinoid syndrome. Excessive serotonin production in neuroendocrine neoplasms consumes tryptophan, a shared precursor for niacin synthesis, ultimately resulting in reduced niacin availability.^6,8,20^ Supplementation with a vitamin B complex containing 20 mg of niacin 3 times daily led to visible clinical improvement prior to surgery.

## CONCLUSION

This case highlights a rare but clinically important presentation of primary ovarian neuroendocrine tumor associated with carcinoid syndrome and carcinoid heart disease. The diagnosis and management were complex and required interdisciplinary collaboration. Preoperative administration of a somatostatin analog played a pivotal role in controlling hormone-mediated symptoms and preventing intraoperative complications. This case underscores the importance of early recognition, biochemical evaluation, and individualized perioperative planning in optimizing outcomes for patients with hormonally active neuroendocrine neoplasms.

## Notes

*This article meets the Accreditation Council for Graduate Medical Education and the American Board of Medical Specialties Maintenance of Certification competencies for Patient Care and Medical Knowledge.*
